# A retrospective single-centre study on the histological profile of brain and spine tumours at a tertiary hospital in Ghana

**DOI:** 10.4314/gmj.v59i1.4

**Published:** 2025-03

**Authors:** Mabel Banson, Felix Siaw-Debrah, Kwadwo Darko, Mawuli K Ametefe, Patrick Bankah

**Affiliations:** 1 Department of Neurosurgery, Korle-Bu Teaching Hospital, Accra, Ghana

**Keywords:** Central Nervous System Tumours, Meningioma, Glioma, Sub-Saharan Africa

## Abstract

**Objective:**

This study aimed to describe the patient demographics, clinical presentation and histological subtypes of central nervous system (CNS) tumours in a tertiary facility

**Design:**

Retrospective review of all the histopathological and medical records available for patients with tumours of the CNS operated on.

**Setting:**

Neurosurgical Unit, Department of Surgery, Korle-Bu Teaching Hospital

**Participants:**

All adult and paediatric patients with histopathologically diagnosed CNS tumours

**Main outcome measures:**

Frequency of histopathological subtypes of CNS tumours

**Results:**

This study of 338 patients with CNS tumours showed a slight female predominance (183 females, 155 males). The mean age was 38.1 years. Brain tumours were more common (290 cases) than spinal tumours (48 cases), with symptoms like headaches (44.44%) and visual disturbance (24.31%) prevalent in brain cases, and paraparesis (35.42%) and low back pain (16.67%) in spinal cases. Certain symptoms were strongly indicative of specific tumour types, such as seizures (OR: 3.3, CI: 1.6 – 6.9, p = 0.005) with meningiomas and visual disturbances with sellar tumours (OR: 6.7, CI: 3.6 – 12.9, p<0.001). Most tumours were low-grade (78.69%). Meningiomas were the most common (33.14%), particularly meningothelial (38.39%). Gliomas, glioneuronal, and neuronal tumours were next in prevalence (28.40%), followed by sellar tumours (18.93%). Astrocytomas (60.42%) were the predominant glioma subtype.

**Conclusion:**

Low-grade tumours predominate in our setting. It is prudent that we channel efforts towards prompt diagnosis and treatment of such cases.

**Funding:**

None declared

## Introduction

Brain and spine tumours represent a significant public health concern due to their potential to cause severe disability and death. The incidence of these tumours has been increasing, underscoring the need for a better understanding of their epidemiology, clinical presentation, and histopathological variations.^1^ Benign tumours typically grow slowly and can often be surgically removed.^2^ In contrast, malignant tumours tend to proliferate rapidly and may spread to other parts of the body. Tumours are also classified as primary, originating from the central nervous system (CNS), or secondary, representing metastases from other organs.^2^ Treatment options for tumours may include surgery, radiation therapy, chemotherapy, or a combination of these methods.^3^

The age-standardized incidence of primary malignant intracranial tumours worldwide is approximately 3.7 per 100,000 for males and 2.6 per 100,000 for females annually.^4^ There is, however, a notable lack of comprehensive reporting on the epidemiology of intracranial tumours in Africa. The incidence in African countries is estimated to be lower than that in more developed countries^5^, yet these estimates exhibit considerable variation across different African regions and countries. Brain tumours are a leading cause of cancer-related mortality and morbidity in children.^6^ At the Korle-Bu Teaching Hospital, for example, the neurosurgical clinic reports an average of two new cases of intracranial tumours weekly, in addition to those presenting in the emergency room.^7^

Additionally, data are scarce regarding the types of intracranial tumours most observed in African countries. Research indicates that meningiomas are the most prevalent type of intracranial tumour among individuals of African descent.^8^

Brain and spinal cord tumours are the fifth leading cause of cancer death in Africa.^9^ It is essential to acknowledge the efforts of various institutions across Africa in reporting the epidemiology of brain and spine tumours. Despite these efforts, the precise incidence of intracranial tumours in sub-Saharan Africa remains poorly understood, primarily due to the inadequacy and unreliability of available data. This knowledge gap adversely affects health budgeting and planning in the region, leading to suboptimal healthcare strategies and resource allocation.^7^

This study aims to detail the demographics, clinical presentation, topography, and histological characteristics of patients presenting to the Korle-Bu Teaching Hospital (KBTH) with CNS tumours. By analysing data from these patients, the objective was to address the lack of population-based data and establish the prevalence patterns of CNS tumours within our institution. Given that KBTH is the leading neurosurgical centre in Ghana, receiving most referrals, this information may provide valuable insights and assist in effecting policy changes in the hospital, applicable to the broader Ghanaian population. Preliminary results of this retrospective study were presented as an oral presentation at the 2023 SNO-SSA Annual Scientific Meeting on July 20, 2023.

## Methods

### Data Collection

This retrospective study was conducted at the Neurosurgical Unit of Korle-Bu Teaching Hospital, Accra, Ghana, spanning 16 years from January 2007 to August 2023. Ethical clearance was obtained from the Ethical and Protocol Review Committee of the Korle-Bu Teaching Hospital (KBTH-IRB 000150/2023). The study involved patients who underwent neurosurgical operative interventions, with histology performed on all extracted specimens for final histopathological diagnosis. All patients were initially evaluated with CT scans, and the majority also had MRI scans. Data collected included patient demographics (name, age, and gender), presenting signs and symptoms, WHO grading, and histological subtypes of tumours, sourced from hospital records. Patients without histopathology diagnoses were excluded from the study. Due to the retrospective nature and the minimal risk to the patients, informed consent was waived by the IRB.

### Data Analysis

We conducted a comprehensive analysis of a dataset comprising various tumour subtypes and clinical features. Initially, the data was examined for quality and consistency, with appropriate handling of missing or anomalous values. We then performed a series of statistical analyses using R Studio (Version 4.3) for data manipulation and statistical testing. The age distribution among all patients, particularly within adult and paediatric groups, was assessed, calculating the mean, standard deviation, and other relevant statistical measures. For gender analysis, we calculated the frequencies of tumour subtypes among male and female patients, employing chi-squared tests to determine any statistically significant differences in tumour subtype prevalence between genders. Furthermore, we explored the association between clinical features and tumour grades by calculating odds ratios and confidence intervals to identify statistically significant relationships. Graphs were created with R Studio (Version 4.3) using the *ggplot* package.^10^

## Results

### Demographics, clinical features and histological classification of patients with CNS tumours

A total of 338 patients were analysed over the 16 years. Of these patients, 44.86% (n=155) were males and 54.14 (n=183) were females. The overall mean age was 38.10 ± 20.20 years, with a range of 1-92 years. Among 267 adult patients, the mean age was 45.79 ± 14.65 years and in 68 paediatric patients, 7.89 ± 4.54 years. Table 1 details the demographics of all patients included in the study.

**Table 1:** Demographics and tumour grading of patients

Variable	Total (n=338) Frequency n (%)	Brain Tumours (n=290) Frequency n (%)	Spine Tumours (n=48) Frequency n (%)
**Male**	155 (45.86)	134 (46.2)	21 (43.8)
**Female**	183 (54.14)	156 (53.8)	27 (56.2)
**Mean Age in Years (SD)**	38.10 (20.20)	37.63 (20.19)	40.87 (20.25)
**Adult Mean Age in Years (n=267)**	45.79 (14.65)	45.58 (14.38)	46.95 (16.21)
**Pediatric Mean Age in Years (n=68)**	7.89 (4.54)	7.56 (4.57)	10.37 (3.58)
**Tumour Grading**			
**Low Grade**	266 (78.69)	229 (78.96)	37 (77.08)
**High Grade**	72 (21.30)	61 (21.03)	11 (22.92)
**WHO Tumour Classification (n=216)**			
**WHO 1**	135 (62.50)	113 (59.79)	22 (81.48)
**WHO 2**	39 (18.06)	34 (17.99)	5 (18.52)
**WHO 3**	12 (5.56)	12 (6.35)	0 (0)
**WHO 4**	30 (13.89)	30 (15.87)	0 (0)
**Tumour Localization**			
**Cervical**	10 (20.83)	NA	10 (20.83)
**Thoracic**	26 (54.16)	NA	26 (54.16)
**Lumbar**	12 (25.0)	NA	12 (25.0)
**Supratentorial**	229 (63.96)	229 (79.0)	NA
**Infratentorial**	61 (18.05)	61 (21.0)	NA

**Abbreviations:** NA: Not Applicable, SD: Standard Deviation, WHO: World Health Organisation

**Figure 1A:**
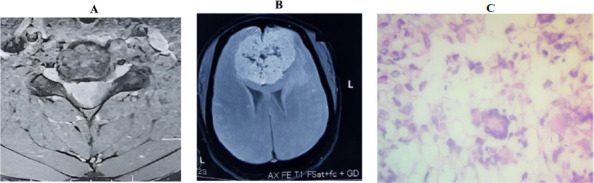
A right-sided, well-defined, moderately enhancing intradural extramedullary mass at the C5-6 level. The mass, measuring 1.9 cm x 1.5 cm x 1.0 cm, appeared T1 isointense and T2 hyperintense, extending to the C6 nerve root and causing severe displacement and compression of the cervical cord. **Figure 1B**. A large, well-defined olfactory groove tumour with multiple areas of calcification, T1 hypointense, and T2 showing avid enhancement on contrast. **Figure 1C**. High-grade glioma with giant cells and markedly pleomorphic hyperchromatic cells with variably textured Stroma. Glioblastoma Multiforme

Of the cases examined, 85.79% (n=290) involved the brain while 14.21% (n=48) involved the spine (Figure 1). The most frequent clinical features observed in brain tumour cases were headaches (44.44%), visual disturbance (24.31%), seizures (13.54%), and hemiparesis (10.42%). In spine tumour cases, the predominant clinical features were paraparesis (35.42%), low back pain (16.67%), bladder dysfunction (14.58%), and abnormal gait (10.42%). Among spine tumour cases, the majority, 54.16% (n=26), were in the thoracic spinal cord (see Table 1).

**Figure 2A:**
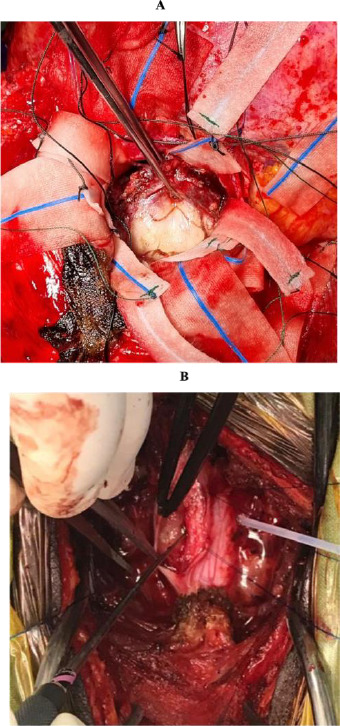
Olfactory groove tumour, moderately vascular and compressing the inferior frontal lobe. A bifrontal craniotomy and bilateral orbital osteotomy were performed, followed by subfrontal excision, achieving a Simpson grade II resection. Figure 2B. A firm, fibrous tumour engulfing the right C6 nerve root. The patient underwent a C4-6 laminectomy and C4-6 posterior cervical lateral mass fusion, including durotomy and complete excision of the tumour. The histopathological diagnosis was a psammomatous meningioma, WHO Grade 1

Most of the identified tumours were low grade, accounting for 78.69% (n=266) of the cases. Among the 216 tumours with a reported WHO Tumour Classification, WHO grade 1 tumour constituted the majority, representing 62.50% (n=135) of the cases. Meningiomas emerged as the most prevalent tumour subtype in this study, accounting for 112 cases (33.14%) among both brain and spine cases. Specifically, there were 97 cases of intracranial meningiomas and 15 of spinal cord meningiomas. Within meningiomas, meningothelial meningiomas were the most common subtype, constituting 38.39% (43/112) of cases, followed by psammomatous meningiomas at 10.71% (12/112) (see Table 4). The second most prevalent tumour histology was gliomas, glioneuronal tumours, and neuronal tumours, which accounted for 97 cases (28.70%) (Figure 2). Sellar tumours were the next most common, with 64 cases (18.93%). Table 4 details the histopathological classification of CNS tumours.

**Table 4:** Histopathological classification of CNS tumours

Histopathological Classification	Total (n=338)	Brain Tumours (n=290)	Spine Tumours (n=48)
**Meningiomas**	112 (33.14)	97 (33.45)	15 (31.25)
**Meningothelial**	43 (38.39)	40 (41.24)	3 (20)
**Psammomatous**	12 (10.71)	4 (4.12)	8 (53.33)
**Atypical**	11 (9.82)	11 (11.34)	0 (0)
**Fibroblastic**	8 (7.14)	8 (8.25)	0 (0)
**Angiomatous**	7 (6.25)	6 (6.19)	1 (6.67)
**Transitional**	6 (5.36)	6 (6.19)	0 (0)
**Anaplastic**	1 (0.89)	1 (1.03)	0 (0)
**Cystic**	1 (0.89)	1 (1.03)	0 (0)
**Microcytic**	1 (0.89)	1 (1.03)	0 (0)
**Papillary**	1 (0.89)	1 (1.03)	0 (0)
**Secretory**	1 (0.89)	1 (1.03)	0 (0)
**Unclassified**	20 (17.86)	17 (17.53)	3 (20)
**Gliomas, Glioneuronal and Neuronal Tumours**	97 (28.70)	92 (31.72)	5 (10.41)
**Astrocytoma**	58 (59.80)	55 (59.78)	3 (60.0)
**Glioblastoma**	22 (22.68)	22 (23.91)	0 (0)
**Ependydoma**	10 (10.30)	8 (8.70)	2 (40.0)
**Oligodendroglioma**	7 (7.22)	7 (7.61)	0 (0)
**Tumours of the Sellar Region**	64 (18.93)	64 (18.93)	0 (0)
**Pituitary Adenoma**	59 (92.19)	59 (92.19)	0 (0)
**Craniopharyngioma**	5 (7.81)	5 (7.81)	0 (0)
**Cranial and Paraspinal Nerve Tumours**	18 (5.33)	4 (1.38)	14 (29.17)
**Schwannoma**	15 (83.33)	4 (100.0)	11 (78.57)
**Neurofibroma**	3 (16.67)	0 (0)	3 (21.43)
**Mesenchymal Non Meningothelial Tumours**	11 (3.25)	7 (2.41)	4 (8.33)
**Chondrosarcoma**	3/11 (27.27)	2 (28.57)	1 (25)
**Hemangioma**	3/11 (27.27)	1 (14.29)	2 (50)
**Hemangioblastoma**	3/11 (27.27)	3 (42.86)	0 (0)
**Epitheloid**	2/11 (18.18)	1 (14.29)	1 (25)
**Embryonal Tumours**	11 (3.25)	11 (3.79)	0 (0)
**Medulloblastoma**	9 (81.82)	9 (81.82)	0 (0)
**Neuroblastoma**	2 (18.18)	2 (18.18)	0 (0)
Haematolymphoid Tumours*	1 (0.29)	1 (0.34)	0 (0)
Choroid Plexus Tumours*	3 (0.89)	3 (1.03)	0 (0)
Pineal Tumours*	1 (0.29)	1 (0.34)	0 (0)
Metastases To The CNS*	20 (5.92)	11 (3.79)	9 (18.75)

* *Haematolymphoid tumours include CNS lymphoma; choroid plexus tumours include choroid plexus papilloma; pineal tumours include pineoblastoma and pineocytoma. Metastases to the CNS include secondaries from prostate, lung, and breast cancers*.

**Abbreviations**

CNS: Central Nervous System

**Figure 3:**
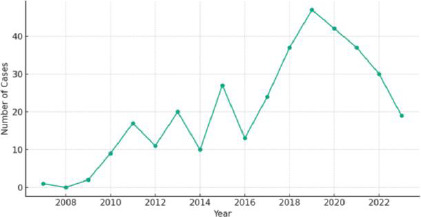
Prevalence of CNS tumour cases (2007-2023)

The average time taken between submission and receipt of histopathology reports was 13.45 ± 29.11 days, ranging from same-day reports to a maximum of 309 days. The trend in case prevalence over the years is illustrated in Figure 3.

## Discussion

In this study, the most frequently reported symptoms in brain tumours were headaches, visual disturbances, and vomiting. Conversely, for spine cases, the most common symptoms were paraparesis, low back pain, and bowel and bladder dysfunction. Seizures were identified as clinical predictors of meningiomas, while visual changes were predictive of tumours in the sellar region. The high incidence of headaches reported highlights their potential role as a cardinal symptom of serious conditions, including brain tumours. A similar pattern of symptoms was observed in a prior study by Ekpene et al^7^ in the same institution. Broader studies on unselected brain tumours in adults have shown that headaches are present in up to 60% of patients.^11^,^12^ However, it's noteworthy that the presentation of a headache as the sole symptom at the time of brain tumour diagnosis is relatively rare, accounting for only up to 8% of patients.^11^,^13^ Typically, most patients present with multiple symptoms. In a study by Vázquez-Barquero et al^13^, all patients with isolated headaches developed additional symptoms within ten weeks of diagnosis. Most brain tumours typically present with headaches due to various underlying mechanisms.^14^ Although presentations can vary depending on the tumour's location, *Wu et al*.^15^ report that meningiomas may cause headaches in up to 50% of patients. The high prevalence of meningiomas could partially explain the reported frequency of headaches in brain tumour cases. *Vikash et al*.^16^ further elaborate on the diverse presentation of meningiomas, noting that in addition to headaches, meningiomas of the occipital lobe convexity, optic nerve sheath, and suprasellar regions can also present with visual loss. Moreover, tumours of the sellar region, which are known to cause visual changes, were identified as the third most prevalent histological subtype in this study. Consequently, the high rate of visual changes observed in brain tumour cases is likely attributable to the significant prevalence of sellar tumours reported. These findings highlight the necessity for a high index of suspicion for early diagnosis and appropriate referral of patients with brain and spine tumours, particularly in those presenting with these symptoms and co-existing red flags, beyond the conventional causes.

In the present analysis, we report that 78.69% of cases had low-grade tumours. Similarly, Garcia et al.^17^ in a study analyzing CNS tumours from 2004-2015 reported that over 60% of tumours were low-grade. *Ostrom et al*.^18^ in their report indicated that the incidence rate of non-malignant brain and other CNS tumours was 17.18 per 100,000, compared to 7.06 per 100,000 for malignant tumors suggesting that benign tumours are more frequently diagnosed. These findings, in conjunction with those observed in this study, highlight that benign tumours constitute most CNS tumours seen in this study. Therefore, efforts should be focused on early diagnosis and appropriate management to improve morbidity and mortality among affected patients.

There is a higher prevalence of CNS tumours in females, with meningiomas being the most commonly reported subtype, accounting for 33.14% of all cases. Several studies have observed gender disparities in CNS tumour incidence, with some reporting a higher occurrence in females^7^,^8^,^19^, while others have noted a greater prevalence in males.^1^,^19^ Existing literature, however, has reported higher rates of meningiomas in females compared to males.^8^,^20^ Primary intracranial neoplasms typically include 13-26% meningiomas.^21^ The Central Brain Tumour Registry of the United States (CBTRUS) has reported meningiomas as constituting 40% of all diagnosed brain tumours between 2015 and 2019.^20^ Additionally, despite a low incidence of intracranial tumours in Japan, *Nakamura et al*^22^ also identified meningiomas as the most frequent tumour type. The findings of the present analysis, combined with existing literature, reinforce that meningiomas are the most diagnosed intracranial tumors, particularly in females. Consequently, the higher prevalence of females in this study may be partially attributed to the high incidence of meningiomas observed.

The decline in case prevalence observed after 2019 might be attributed to the COVID-19 pandemic, which likely resulted in decreased hospital visits. This reduction in attendance could be due to various factors, including fear of contracting COVID-19, the implementation of lock-down measures, and the cessation of specific healthcare services.^23^-^25^ Nonetheless, the continuation of this downward trend necessitates further exploration. In the extant literature, numerous definitions for turnaround time (TAT) are used.^26^

TAT can be defined as the “time from receipt of the specimen” until the “time of availability of the result” (laboratory TAT)^27^, as well as the “time from the physician's request” until the “time the physician views the result” (total TAT).^28^ The time it takes to process samples in surgical pathology is an important indicator of quality control.^29^ Studies have found that around 24% of cases do not meet the standard turnaround time set by the College of American Pathologists (CAP), which requires at least 90% of routine cases to be reported and verified within two days.^29^ The average turnaround time was 13.45 ± 29.11 days, far from meeting the CAP standard. Values of 2–16 days were reported in a study done in Kano, Nigeria.^30^ Alshieban and Al-Surimi^29^ described that the stages of slide allocation and delivery to pathologists, slide review by pathologists, report editing by transcriptionists, and report verification by pathologists, are the points at which the majority of delays tend to occur. This value could be skewed by the inclusion of cases dating back to 2007, as older cases often faced numerous diagnostic challenges. However, advancements and the expansion of the field of pathology in the country have significantly improved the diagnostic processes in recent years. Nonetheless, these findings are significant as they greatly influence the diagnosis and subsequent referrals to other specialty units in the continuum of care. Although the reasons for these delays are beyond the scope of this study, it is crucial to focus on this process to prevent undue delays and streamline the diagnostic procedure.

The retrospective nature of this study, relying solely on retrievable medical records, potentially led to an underestimation of the true prevalence of CNS tumours at the study institution. As such, some cases may have been omitted from the study period. Future research should prioritize morbidity and mortality associated with CNS tumours. Comprehensive, prospective, multicentre studies of longer duration across various institutes in Ghana are necessary to gain a more accurate understanding of the prevalence and incidence of CNS tumours. The establishment of an institutional registry for CNS tumours could serve as a valuable platform for aggregating comprehensive data, this pilot could extend to other neurosurgical centres in the country. Such an initiative would offer critical insights into the epidemiology of CNS tumours in the population and aid in the development of effective prevention, diagnosis and management strategies.

## Conclusion

This study was conducted to delineate the spectrum of CNS tumours in a tertiary institution in Ghana. The results identified meningiomas and low-grade gliomas as the most prevalent tumours. Additionally, the study revealed that sample turnaround times did not match the standards set by the CAP, highlighting the need for a detailed investigation of subprocesses to identify delays, optimize sample flow and reduce reporting times. Low grade tumours predominate in this setting, and it is prudent to channel efforts towards prompt diagnosis and treatment of such cases.

## Figures and Tables

**Table 2:** Frequency of clinical symptoms and signs in brain cases

Clinical Sign/Symptom	Frequency n (%)
**Headaches**	128 (44.14)
**Visual Disturbance**	70 (24.14)
**Seizures**	39 (13.45)
**Hemiparesis**	30 (10.34)
**Gait Disturbance**	20 (6.90)
**Vomiting**	19 (6.55)
**Altered Sensorium**	15 (5.17)
**Cerebellar Signs**	8 (2.76)
**Proptosis**	8 (2.76)
**Dysphasia**	4 (1.38)
**Skull Swelling**	4 (1.38)
**Cranial Nerve Palsy**	3 (1.03)
**Macrocephaly**	3 (1.03)
**Hemiplegia**	3 (1.03)
**Hearing Loss**	3 (1.03)
**Ophthalmalgia**	2 (0.69)
**Signs Of Acromegaly**	2 (0.69)

**Table 3:** Frequency of clinical symptoms and signs in spine cases

Clinical Sign/Symptom	Frequency n (%)
**Paraparesis**	17 (35.42)
**Low Back Pain**	8 (16.67)
Bladder Dysfunction*	7 (14.58)
**Gait Disturbance**	5 (10.42)
**Paraplegia**	4 (8.33)
Bowel Dysfunction*	4 (8.33)
**Neurofibromas**	3 (6.25)
**Sensory Deficits**	3 (6.25)
**Neck Pain**	3 (6.25)
**Hemiparesis**	2 (4.17)
**Upper Limb Weakness**	2 (4.17)
**Quadriparesis**	2 (4.17)
**Cafe Au Lait Spots**	2 (4.17)
**Iris Lischs Nodule**	1 (2.08)
**Quadriplegia**	1 (2.08)

* *Bowel and bladder dysfunction includes urinary retention, urinary incontinence, increased urgency, frequent urination, difficulty urinating, constipation, bowel incontinence and changes in bowel habits*
